# Supplementary material for: The Effect of Iron-Fortified Lentils on Blood and Cognitive Status among Adolescent Girls in Bangladesh
Source: Nutrients. 2023 Dec 2;15(23):5001. doi: 10.3390/nu15235001 (PMC10707902; doi:10.3390/nu15235001)
Supplement: Supplementary file 1 [file nutrients-15-05001-s001.zip › nutrients-2695479-supplementary.pdf]

Supplementary information for *Nutrients* ms 2695479
The effect of iron-fortified lentils on blood and cognitive status among adolescent girls in Bangladesh

Methodological details, cognitive measures

Simple reaction time (SRT) task: Participants were instructed to press a key as soon a simple visual stimulus (a single character) was presented on the computer monitor. Five practice trials were completed in which feedback was provided. Following the practice trials, there were 20 test trials and feedback was not provided. The interstimulus interval varied on each trial with a range of 750-1500 ms.

Go/no-go (GNG) task: Participants were presented two stimuli in random order, one that they were supposed to respond to ('go') and one to which they were supposed to inhibit their response ('no-go'). On 20% of the trials, the 'go' stimulus was presented while on 80% of the trials the 'no-go' stimulus was presented. Participants responded to the 'go' stimulus by pressing the '/' key with the index finger of their dominant hand. Ten practice trials were completed prior to testing and participants were provided feedback on the accuracy of their responses. Following this, a total of 30 'go' trials and 120 'no-go' trials were completed without feedback.

Attentional network task (ANT): Trials of this task began with the presentation of a central fixation cross followed by a cue (an asterisk) in one of four conditions: 0 cue (no asterisk), 2 cues (one asterisk above and one below fixation), center cue (one asterisk at fixation), or a spatial cue (one asterisk either above or below fixation). The spatial cues were always valid, in that they always predicted the location of the arrows to be judged. The cue was followed by the test display, consisting of a central arrow either above or below fixation pointing either right or left, flanked by two elements on the left and the right side of the central arrow. The flanking elements could be in one of three conditions: congruent (arrows pointing the same direction as the central arrow), incongruent (arrows pointing the opposite direction of the central arrow), or neutral (line segments without arrow heads). Participants were instructed to judge the direction of the central arrow, ignoring the flankers, using the z or / keys. Reaction times (RTs) to each of the trial types were measured and used to calculate three summary scores. The first was an alerting score, which was calculated by subtracting the median RT (correct responses only) for trials with 2 cues from the median RT for trials with 0 cues. The second was an orienting score, which was calculated by subtracting the RTs for trials with spatial cues from the RTs trials with center cues. The third was a conflict score, which was calculated by subtracting the RTs for trials with consistent flankers from the RTs for trials with inconsistent flankers.

Sternberg memory search (SMS) task: On each trial, participants were presented with a set of either 1, 3, or 6 items (graphics characters) to be remembered. Participants were then presented with a test item that was either in the presented set (an old item) or that was new. RTs for old and new items are separately regressed onto set size, giving an intercept and a slope for searching for each type of item for each participant. The intercept measures the speed with which memory can be accessed and the slope measures the rate at which memory can be searched.

Cued recognition task: Participants studied 24 items and were tested on the old items and 24 new items, randomly presented. At test, the items were presented with two, three, or four quadrants (two, three, or four cues) of the image visible, and judged the image by pressing the one key for a “new” judgment and another key for an “old” judgment. No feedback was provided. The median RT for correct responses to old and new items at the 4-cue level was calculated for each participant. In addition, we fit the Cox proportional hazards model to individual participants RTs to model the reductions in RT as function of number of cues. The magnitude of the improvement in RT with increases in the number of cues can be calculated from the β value estimated by the proportional hazards model as

$$\left(e^{\beta} - 1\right) \times 100\%$$

This value can be interpreted as the percent increase in retrieval capacity due to additional cues.

Supplementary Table S1: Changes in the cognitive variables as a function of response status for Hb. Entries for the three categories of response status are the mean change in the variable from BL to EL.

					Least Squares Means (SE)			
Task	Change variable	*F*	*MSE*	*p*	Decrease (D)	No Change (N)	Increase (I)	Ordering
SRT	Mean RT (ms)	2.44	1161	0.089	1 (3)	-1 (3)	-12 (5)	I < N = D
GNG	Mean RT (ms)	0.92	1096	0.399	4 (3)	0 (3)	7 (5)	I = N = D
ANT	RT 0 cues (ms)	0.63	7877	0.534	-39 (7)	-43 (8)	-24 (14)	I = N = D
	RT 2 cues (ms)	2.70	10024	0.068	-23 (8)	-51 (9)	-28 (16)	I = N = D
	Alerting (ms)	1.77	11483	0.172	-15 (9)	8 (10)	4 (17)	I = N = D
	RT center cues (ms)	0.22	6972	0.801	-53 (7)	-47 (8)	-45 (13)	I = N = D
	RT spatial cues (ms)	1.84	9566	0.161	-5 (8)	-27 (9)	-9 (16)	I = N = D
	Orienting (ms)	3.30	7883	0.038	-48 (7)	-20 (8)	-36 (14)	I = N = D
	RT incongruent flankers (ms)	0.12	11452	0.884	14 (9)	8 (10)	6 (17)	I = N = D
	RT congruent flankers (ms)	1.85	14223	0.159	-10 (10)	-37 (11)	-13 (19)	I = N = D
	Conflict (ms)	1.02	18151	0.363	24 (11)	45 (13)	19 (22)	I = N = D
SMS	Intercept, new items (ms)	0.05	116536	0.954	32 (27)	19 (31)	25 (51)	I = N = D
	Slope, new items (ms/item)	0.45	3714	0.639	21 (5)	14 (5)	14 (9)	I = N = D
	Intercept, old items (ms)	0.48	47049	0.619	-4 (17)	6 (19)	32 (33)	I = N = D
	Slope, old items (ms/item)	0.07	785	0.929	12 (2)	13 (3)	13 (4)	I = N = D
CRT	RT new items (ms)	0.29	28515	0.750	-21 (14)	-31 (16)	-9 (27)	I = N = D
	RT old items (ms)	1.86	26846	0.157	-4 (13)	-26 (15)	32 (26)	I = N = D
	Percent change in capacity (%)	0.67	2270	0.514	-12 (4)	-8 (5)	-3 (8)	I = N = D

Note: SRT = simple reaction time task, GNG = go/no-go task, ANT = attentional network task, SMS = Sternberg memory search task, CRT = cued recognition task, RT = reaction time, Neg. = negative, MSE = mean square error.

Supplementary Figure S1: Proportion of the participants at each age who reported having reached menarche at BL and EL.

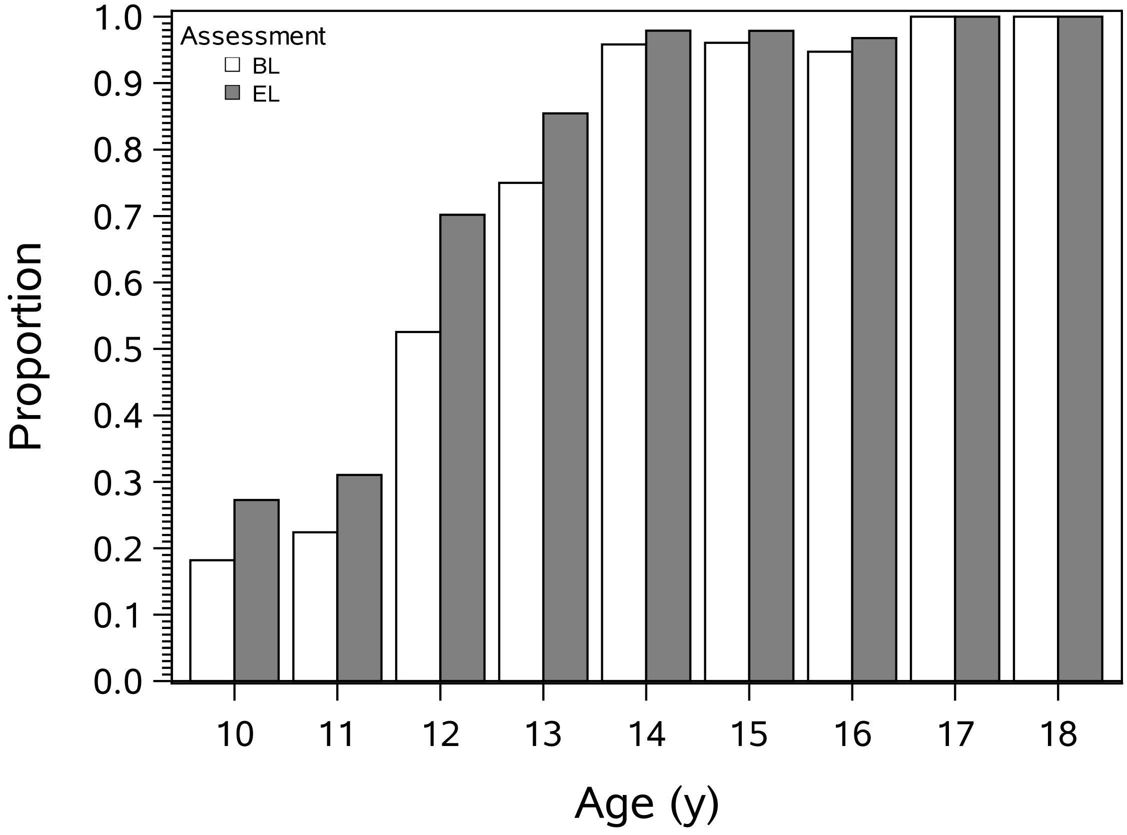

Supplementary Figure S2: Changes (BL to EL) in (a) Hb, (b) sFt, and (c) TBI for all participants in the cognitive assessments.

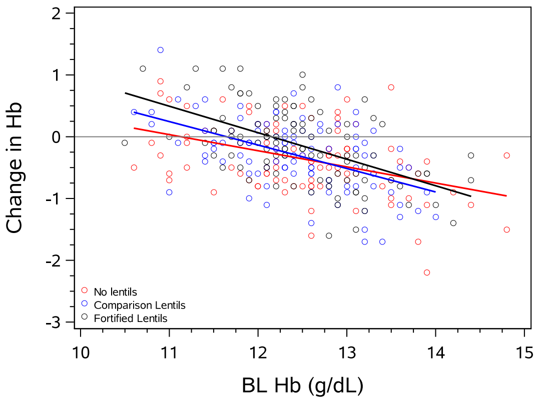

(a)

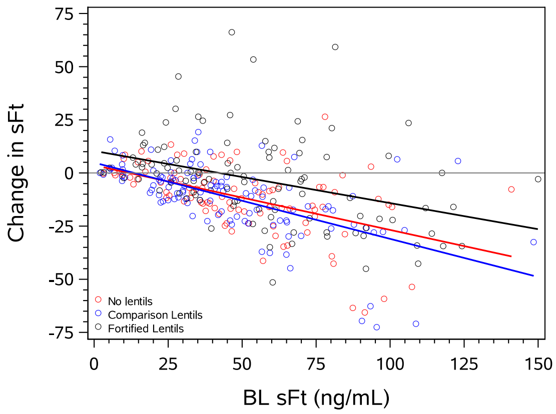

(b)

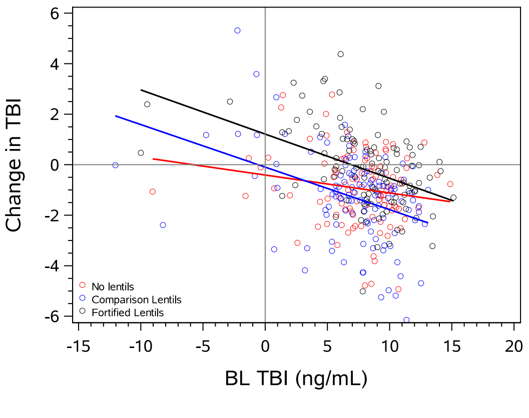

(c)
